# Extracts

**Published:** 1882-06

**Authors:** 


### Extracts.

MEDICINE.—An inaugural thesis presented to the faculty of the Missouri Medical College:

To calm the woes of human kind,
The men of philanthropic mind
For ages past, have been inclined
With great consideration.
That they have failed, to some extent,
Can be no great disparagement;
For, ever with the best intent,
They have adorned their station;
And, to the midst of pain and woe,
Meet for faint mortals here below,
Brought succor and a gladsome glow
Of peace and consolation.

A glorious mission has been theirs,
Through the long line of passing years,
Out of our sight, 'mid smiles and tears,
Forever drifting, drifting.
Now joy and hope, now grief and woe,
Around, above; we see them go
With steady step, serene and slow,
As life's strange scenes are shifting.
On, on and up, toward the goal
Where glory waits the faithful soul,
When our great earth shall cease to roll
And morning is uplifting.

A glorious mission is our own;
More glorious than the world has known;
For we have largely overgrown
    The stature of *their* learning.
But, if our ardor would be blest,
Each should contain within his breast
A strong desire, a vague unrest,
    A constant, mighty yearning.

Each of us has his place to fill
And each can *fill* it, if he will:
Our work will be for good or ill,
    So long as we are living.
Long as our pulses beat and play,
We must press forward on our way
And toil and strive by night and day,
    The woes of man relieving.
Is not our work a glorious one?
Is not the race we have to run
The noblest underneath the sun?
    We labor on, believing.

Where moves the battle's martial line,
And bloody sabers flash and shine,
And wreaths of powder-smoke entwine
    The columns rent and gory;
When, from brave hearts, with plash and spout,
The crimson life-blood gushes out,
And thousands, trampled by the rout,
    Lie on the field of glory;
The surgeon with his skillful brain
And gentle hand relieving pain,
Seems as one sent from Heaven's train,
    To close the dismal story.

When, gliding swift from strand to strand,
With ghastly face and icy hand,
The pestilence pervades the land;
    So slyly, surely creeping
Upon its victims; and the wail,
The frozen hearts, the cheeks all pale,

The souls that feebly quake and quail,
 The grief and woe and weeping
Bespeak that death, grim foe, is near;
And that his victims, on the bier,
Or in the earth's embrace so drear,
 Eternally are sleeping:
Then our brave brethren, bless them all!
Stand round the poison-breathing pall,
Where they, alas! too often fall,
 Their faithful vigils keeping.

In the lone sick-room, when the heart
Beats slowly, feebly, and the dart
Of death is raised to strike apart
 The soul and frame forever;
When the death-damp stands on the brow,
And the faint breath comes weak and slow
And the dim eye is glazing, oh!
 How strong hearts shake and quiver.
But the physician, cool and calm,
Whose presence gives each bruise a balm,
Stands by to watch, without a qualm,
 The spirit cross the river.

No longer talk of heroes proud,
Who wake the earth with thunder loud,
Whose fame rides in a glowing cloud,
 Where wrath and strife are gleaming.
No longer prate of those whose bands
Waste cities fair and fruitful lands
In fell destruction, and whose hands
 With crimson blood is streaming.
He, who serenely dies to save
His fellow man, is far more brave,
And rests within a *nobler* grave;
 Though humbler far its seeming.

Such are the men whose deeds are wrought
For feeble honor, dearly bought;
Whose path through life is daily fraught
 With danger and privation.

Such is the work, brave work and true,
That stretches out before our view,
And is entrusted to the few
Of our beloved profession.
Such the rewards, which are to be
Bestowed on us, if only we
Will labor conscientiously
To fill our situation.

—*St Louis Med. and Surg. Journal.*

---

Aseptic Vaccine Lymph.—Much practical interest attaches to the subject treated in the *Jahrbuch fur Kinderheilkunde*, Neue Folge, Band xvii, p. 172, by Dr. R. Pott, who gives the results obtained by him in experimenting with vaccine lymph modified by combination with salicylic, boracic and carbolic acids. The mixture employed consists of salicylic solution (1 to 300), boracic acid solution (3.5 per cent ), and carbolic acid solution (1 to 5 per cent.), each of these being combined with an equal part of humanized lymph. All proved active except the 5 per cent. carbolic acid solution. This result corresponds with that obtained by Dr. Braidwood and Mr. Vacher in their more extended and carefully prosecuted experiments detailed in their second and third reports to the Scientific Grants Committee of the British Medical Association. We do not learn from Dr. Pott his method of procedure, the amount of care he bestowed on testing the inoculative efficacy of the vaccine he used, his manner of mixing it with the antiseptic solution, the length of time during which the vaccine was exposed to the antiseptic influences before inoculation, the cleanliness of the instruments he used, and other such details. On the other hand, Braidwood and Mr. Vacher have insisted on the great importance of using special instruments for each set of experiments; on the necessity of testing the efficacy of the vaccine itself before drawing inferences from its action when mixed with antiseptic solutions; on the influence of lengthened contact with the antiseptic, especially in the case of carbolic acid; and on the falsity connected with the ordinary manner of conducting such experiments by inoculating a subject on the one arm with vaccine, and on the other with the experimental fluid. In their second report they state "that a saturated solution of salicylic acid does not impair the efficacy of the vaccine contagium;" and that a saturated solution of boracic acid "impairs the vitality of vaccine contagium little if at all, even after having been kept some days in contact with the contagium particles." On the other hand (in their first report), their conclusions in regard to the influence of carbolic acid on the vitality were "(*a*) that the immediate inoculation of a mixture of vaccine and a moderately strong solution (1 to 20 aq.) of carbolic acid succeeds in a certain number of instances; (*b*) that such a mixture, preserved for some time, seventeen days to six weeks, fails to produce vesicles;" and that the mixture of vaccine with stronger solutions of the acid, and likewise with carbolate of glycerine "destroys the efficacy of vaccine." They have further proven that antiseptics in the gaseous state are much more potent destroyers of the vitality of vaccine than are such in solution or in fluid form. Dr. R. Pott claims the following advantages for these aseptic lymphs: 1. The "erysipelatous poison" contained in the lymph is probably destroyed, and the early vaccinal erysipelas in this way prevented; 2. Such lymph may be kept for years without spoiling; 3. The lymph is thinner, and contains no fibrinous coagula. To us these inferences appear strange and illogical. We are not aware that the "erysipelatous poison" has yet been demonstrated; but we know that Jenner laid down the axiom (and the every-day experience of public vaccinators in England has proved its correctness) that *no* areola should surround the vesicles on the eighth day, and that the redness which appears on the ninth to the eleventh days after vaccination should be evanescent. When post vaccinal erysipelas occurs, this is not to be ascribed to the vaccine, but to its admixture with blood, epithelium, or impurities from the skin; or to the employment of uncleansed instruments for the operation; occasionally to defective sanitary arrangements. That vaccine may, by the aid of these antiseptics, retain its vitality "for years" we very much doubt On the other hand, Dr. W. Husband (in his essay on the subject) states that vaccine, properly secured in his capillary tubes, has been known to retain its efficacy for "seven years." Lastly, if a vaccinator desires to render his lymph thinner, and to free it from fibrinous coagula, he has merely to dilute it with glycerine, or with a mixture of glycerine and water, in order to obtain his desire. This last form of commixture, we are informed, is frequently carried out by the public vaccinator in Berlin, who considers that by this means he renders his vaccine lymph more pure.—*British Med. Jour.*—*Med. News.*

---

Physiology of the Ox, and the Question of Beef-Cooking.—An ox lay stretched on a long table in the "demonstration hall" of the College of Pharmacy, at Twenty-third Street and Third Avenue, Tuesday afternoon. The ox was dead and his skin was off, likewise his head and his tail and his hoofs. Behind the table stood a lady well known in cooking circles. It was Miss Parloa, author of any number of books on cooking and household management and marketing, and she was there to lecture on marketing. Beside her, also behind the ox, stood a butcher. He was neither big nor burly, but he wore an apron about his body and an expression of determination on his face as he looked at the ox. He had already cut the ox in halves and in quarters. Before the table on the long benches where studious druggists are wont to sit sat about one hundred ladies. They were accompanied by four gentlemen. The ladies wore sealskin cloaks and carried note books and pencils in their hands. Some of them had little pictures of an ox, which they compared with the original on the table.

Miss Parloa began to talk, and the pencils flew over the little note books. "This is fat," remarked the lecturess, patting a certain portion of the ox that was of a creamy color, with streaks of dark red running through. The ladies wrote "fat" and the butcher nodded approvingly. "This is suet," continued the speaker, taking up a piece of a still whiter substance and crumbling it in her hand, "and it is almost a sure sign of good beef when the suet crumbles nicely." One hundred little notes went into one hundred little books, and one hundred family butchers will be made miserable by the application of the suet test to beef. In the meantime the butcher stood behind the ox and smiled in a sort of a patronizing way.

The lecturer was quite positive that exercise was an excellent thing for an ox. People who took plenty of exercise were generally the most healthy, and what was true of a man or a woman was true of an ox. Exercise invigorated the tissues, fibres and muscles of the body, and sent the blood coursing through the system. Where-ever the blood went some of it stayed and enriched the fibres and tissues. This was particularly so of an ox. At this point the butcher created a little diversion by nearly stabbing himself in the eye with a long knife. When order had been restored the audience grasped their pencils once more and Miss Parloa calmly continued the question of exercise. It was not necessary, of course, that an ox should be entered into a six-day go-as-you-please before entering the shambles, nor was it advisable that he be turned loose in an up-town street to afford policemen an opportunity of opening a fusillade on inoffensive people, but the fair speaker was decidedly of the opinion that no ox was complete without exercise. From the time of the first ox it was a recognized fact among all students of physiology that the muscles most used were the most healthy.

"The trouble with New Yorkers is," said Miss Parloa, "they all want the best cuts."

"Why; so they do," whispered several of the pupils, looking at each other with an air of surprise. The butcher nodded and grinned. He was at home once more.

"No matter how poor the buyers are they want the very best. That is why beef is generally so dear."

"How very clever!" said one of the ladies in the fourth row, and there was general applauding.

"Now," continued the preceptress, "most people consider the tenderloin as the choicest portion of the beef; certainly they pay the most for it. As a matter of fact, it is anything but the best part; it isn't even a good part.

It has no flavor and very little nutrition. Am I not right?" to the butcher.

"I wouldn't eat it, ma'am," he replied, with an air of disgust, and the audience laughed.

"No doctor who cared to give his patients nourishment would order the tenderloin. If he only wanted to fill them up he might order it, but not otherwise."

"What is the best part of the beef, please?" said a trembling voice.

"The toughest portions are the best," replied the speaker, smilingly, but with emphasis.

The hind quarter of an ox was then carved. The butcher cut and Miss Parloa talked. Boston butchers usually cut the hind quarters a little longer than the New Yorkers, but the speaker was not sure but the latter were correct. Then the ribs were turned over and the inside shown. "This is the tenderloin," said the speaker. "It commences here," pointing to a small spot about an inch wide on the fore quarter, "and runs back here on the hind quarter until it reaches its thickest part, behind this fat."

"Runs into the ribs," suggested an auditor.

"Yes; but we do not call them ribs," replied the anatomist.

"Oh!" rejoined the questioner, as she subsided.

"The round, or rump, or neck is one of the best portions of the beef, though the sirloin, or as you call it the porter-house, is the best for roasting. It is not cut right however," continued Miss Parloa, who then proceeded to tell how it should be cut. During this explanation she said that the small end of the loin was the juciest part of the beef, and immediately all of the ladies wanted the small end of the loin pointed out, which was done. The expert butcher then cut out the "flank steak," "inner" and "outer," and the lecturess said that a most delicious dish might be made from them to serve cold. The steaks were to be rolled up with some dry dressing, such as bread or crackers, seasoned to taste and tied, then boiled.

"The flank is only twelve cents a pound," said the butcher, whereupon all hands and the lecturer laughed.

"The trouble with us is that we are too fast. The Americans are a fast people, and want to do everything in a minute. Meats should be boiled five hours—not boiled either, but only simmered. The water should only just cover the meat and never boil—it should simmer only. They do this thing better in France, possibly because fuel is dear."

"Will that bone," pointing to the knuckle shining in its red cushion of beef, "make the best soup?" asked a lady.

"Meat makes the best soup," said the butcher.

This not being denied by the fair lecturer, being satisfactory to the butcher, and the most intelligible part of the proceedings, the meeting adjourned *sine die.*—*Gaillard's Med. Journal.*

---

The Treatment of Eczema.—The patient complains of a burning heat, there is considerable swelling of the affected part, and sometimes the formation of innumerable vesicles. Let alone or treated only by soothing remedies, the swelling goes down, heat disappears, and a slight desquamation alone remains.

Poultices frequently used generally do harm in this stage; the warmth does not relieve the irritation of the peripheral termination of the nerves, and the external irritation of the poultice often extends the disease. The best means at our disposal is the use of soothing lotions, and of these I know none better than one containing two grains of acetate of lead and five minims of dilute hydrocyanic acid to the ounce of distilled water. Soft linen rags must be dipped in the lotion, applied to the part and changed as often as they become dry. Cold water rags may be used in the same way, but it must not be attempted to keep the linen moist by covering it with oil silk; for by so doing the application soon becomes a warm one. And here I would remark on the great importance of using soft rain or river water, or distilled water, in the making of lotions—the salts in the hard water are themselves irritants, and aggravate the malady.

Acute eczema then generally improves, hyperæmia disappears, and perhaps a little desquamation alone results to tell of its presence, but sometimes this is not the case. The disease passes from the acute to the chronic condition, we no longer have burning heat, but an intolerable itching takes its place, aggravated by warmth, whether from contiguity to the fire or the heat of the bed at night, owing to the induced dilatation of the cutaneous capillaries and irritation of the nerve filaments. Vesicles are formed; or some of the epidermic roofs break down, moistening the surface of the skin with an albuminous exudation; in some pus takes the place of serum and we get a crop of pustules which by bursting leave scabs and crusts.

Owing to the excited scratching the disease is extended, and in other parts erythema is set up and papules are formed. So here we have, as the result of an acute eczema, scales, crusts, vesicles, pustules, papules and erythema. Are we now to describe the secondary disease as a case of E. erythematodes, E. impetignoides, E. veticulosum, or E. lichenoides. I think it is obvioüs such a description would be satisfactory to none. The picture I have drawn is a very common one, and furnishes to my mind a very strong argument for a diminution of our dermatological phraseology.

Crusts and scabs do not occur to any great extent on non-hairy parts of the body, as they are rubbed off by contact with external objects, but they cling to the hairs on the head or face, and constitute a serious difficulty in treatment. They must be removed, and this point must be insisted on to our patients who, from lack of industry, deny the possibility of removal. I generally order the head to be anointed with any oil, sweet or almond oil being preferable, and a flannel cap to be worn day and night until the crusts are sufficiently softened to be removable by the fingers or a comb. Should this means fail, a poultice will always effect our object. Cutting off the hair short is not in the majority of cases necessary, but removal of scabs is a *sine qua non* for success. Having done this, say in a case of eczema capitis, the question of further treatment arises, and we must start by remembering two points of importance; first, that if the air be allowed free access to the discharging surface, crusts will inevitably again form, and the trouble of removal again arise; secondly that frequent washing keeps up the irritation, and soddens the parts so that our ointments do not adapt themselves exactly to the affected places. While we have a condition in which pustulation is going on, ointments should be used of a soothing character, and I know af none better than the unguentum zinci oleatis.

The real point of importance is that the ointment should be used in larger quantities, thickly plastered on, and be an effectual barrier against the admission of air. In these cases, too, a linen night cap should be worn, partly from motives of cleanliness, but mainly with one principal object of air exclusion in view. Under this mode of treatment pustulation soon ceases, scabs no longer form, and the surface heals. If we now examine the head, especially in a scantily covered child's head, we find the skin rough, harsh and papular.

We may discontinue our ointment, and begin to wash the head every other night with Hebra's spiritus saponis kalinus. This is made by mixing two parts of the sapo viridis of the B. P. with one of spir. vini rectificatus; allowing the mixture to stand for twenty-four hours, then filtering and adding to the filtrate a little ol. lavandulæ or other sweet smelling scent. A little of this liquid should be poured on to a flannel previously dipped into warm water and the flannel rubbed on the head until smart lather be caused, care being taken that the liquid does not run into the eyes. The lather should be allowed to remain on the head some ten minutes or so, and then played off with warm water. This should be done at bed time, and in the morning some hair oil should be used, and the case will soon be well.

Before leaving the subject of the head eczema I wish to refer to the well known dependence of it upon the irritation caused by lice; they should always be sought for in a dirty subject and will generally be found, nearly always indeed where we get enlargement of the glands at the back of the neck, co-existing with a slight but very irritable eczema at the foot of the hair behind.

When the hairy part of the face is affected, constituting the sycosis of many authors (I do not wish by this to insinuate there is no such disease as a sycosis caused by a parasite, though I am sure that such a disease is rare), treatment is often very difficult; shaving is, I believe, inadmissible, for one reason it is painful to the patient, for another it keeps up daily a prejudicial irritation, and close cropping of the hair is equally effective for exposing the diseased spots and permitting the application of remedies. Crop the hair then and get to work by removing crusts; apply your ointment diligently.

There is one point I should like to bring before this society, as I believe it can only be settled by the wisdom of many and accumulated experiences. German dermatologists ridicule the idea of a disease being driven in, as they argue that the cure of a skin disease can never cause another malady to appear. Another school argues that a skin disease is often a safety valve preventing a serious explosion. I prefer the first idea, but reluctantly am forced to adopt the second. The idea of vicarious menstruation is not a myth, the occurrence of hæmoptysis after the stopping of a hæmorrhoidal flow is a reality, and few of us have not seen the evils arising from stopping a discharge in a case of laboring heart. The old offensive cry of our dependence on a *post hoc propter hoc* logic is triumphantly raised, but I would protest against the imputation.

It would be impossible for me in the limits of a brief paper to consider either all ointments, all lotions, or the treatment of eczema in every situation. My object has been to formulate, if possible, a system of treatment founded on a due conception of the pathology of the disease, and I will ask your permission to express in a few brief, and I doubt not debatable axioms, my views of the disease and its treatment.

1. Eczema is a catarrh of the skin.

2. Its local manifestation may be erythema, a papule, pustule, or vesicle.

3. It may commence acutely, and tend then to spontaneous recovery or chronicity.

4. When chronic, not only are vesicles, etc., formed, but exudation takes place in the true skin.

5. Such exudation must be removed, which must be by absorption by the medium of the blood-vessels.

6. Hard water must be always avoided in treatment.

7. In all acute conditions lotions do good, ointments do harm.

8. Air should be excluded.

9. Water used but little.

10. Crusts must be removed.—*R. M. Simon, M.R.C.P., Lond., in Birmingham Med. Review.*

---

Topical Use of Iodoform.—Fully cognizant of the fact that, like most new remedies, iodoform has had placed to its credit claims which are in many respects unwarranted, I do not vaunt it as a panacea in all cases requiring topical treatment, but will venture the assertion that as an application in a large variety of cases in which there is a solution of continuity of soft parts it far surpasses all other known remedies and has a wide range of usefulness, being a powerful antiseptic as well as acting as an alterative to diseased surfaces.

With these few preliminary remarks I shall now proceed to the consideration of some of the conditions in which it is applicable.

*Venereal Sores.*—For the past three years my only form of treatment of this class of ulcer has been the application of iodoform in powder, dusted over the diseased surface twice or three times daily, and the results have uniformly been most gratifying. In all such cases, without a single exception, and I have treated quite a number, the cure has been remarkably rapid and I have seen the "soft" sore heal within three weeks, and the hard or true Hunterian chancre, which usually runs a course of from six to eight weeks, I have seen completely cicatrized in four weeks, and sometimes less, under this treatment.

It is not an uncommon occurrence to see venereal ulcers take on a phagedenic aspect. In such cases iodoform is equally efficacious, and, in my opinion, is the only treatment to be pursued.

As a dressing in suppurative buboes, iodoform has no equal, producing, as it does, rapid granulation after the evacuation of the contained pus. Although recommended in solution in collodion as sorbefacient to be painted on inflamed inguinal glands for the purpose of preventing suppuration, I must confess that I have not found it answer my expectations.

In fissure of the os uteri, which frequently happens, especially after first labors, no application is so useful as iodoform. True, many of these get well without any treatment, but we are sometimes called upon to treat cases in which, owing to existing conditions, spontaneous union does not take place and the fissure remains, giving rise to much annoyance.

Until I used iodoform I had experienced much trouble in the treatment of a few cases of this nature which came under my observation, but since I have been using the drug I have seen two cases which got well in ten days under its use.

In erosions of the os or vaginal portion of the cervix, whether due to endo-cervicitis or not, iodoform is the best application. I admit that this condition is frequently maintained by endo-cervical disease, and that by curing the disease the erosions will disappear; but iodoform acts admirably in chronic cervicitis, and doubtless by its influence on this disease hastens the healing of the erosions.

In treating such conditions, I generally apply a tampon of absorbent cotton saturated with a solution of iodoform in glycerine.

I was recently called upon to treat a case of gonorrhœa in a female which was completely cured in six days by the use of hollow suppositories containing iodoform.

As a sorbefacient and anodyne in epididymitis and orchitis, this remedy meets the indications admirably. Under its use the enlarged gland rapidly returns to its normal size.

In syphilitic ulcerations of mucous membranes I have used iodoform with the most happy results.

While discussing this remedy we are reminded of the fact that its odor makes its use very objectionable, and frequently makes us hesitate in using it on that account. Patients always object to its disagreeable, pungent, all-pervading odor, and many have been the devices for rendering it inodorous.

I have as yet failed to find anything which will completely deodorize it. Tannic acid in the proportion of one grain to four of iodoform will partially destroy the odor, but the best agent is coumarin, the alkaloid of the Tonqua bean, a sample of which I here show you. The alkaloid is almost, but not completely a deodorizer. The mixture which I now show you is deodorized, but inasmuch as the alkaloid is more volatile than the iodoform a slight odor of the latter can be detected after exposure to the air for some length of time. One grain of coumarin will deodorize forty grains of iodoform.

However this deodorizer may act when the drug is ap plied externally, when used as an intravaginal application nothing will prevent emanation of the odor, for it is very rapidly absorbed, and while most of it is given off by the urine a small portion is exhaled by the skin and lungs, and of course is very soon perceptible. Therefore, when used otherwise than externally no advantage is gained by deodorizing the drug with compounds which possibly may exercise a deleterious influence upon its efficacy.—*L. F. Salomon, M.D., in N. O. Med. and Surg. Journal.*

---

ELECTROLYSIS IN THE TREATMENT OF AORTIC ANEURISM.—At this point let us discuss the treatment of internal aneurisms. As they are internal and not in reach of the surgeon, so that the main arterial trunk may be ligated, the following medical means have been thought of:

First.—Specific remedies, and those that will favor clotting of blood, as the mineral salts, lead, etc.

Second.—Physicians finding that, as people were starved, the blood became very rich in fibrin, thought that there was more likelihood of a clot being formed under a plan of partial starvation than when the patient was allowed full diet.

Third.—As all increased muscular exertion causes increased action of the heart, and hence, the more immediate danger of rupture by the increased force of the blood current through the sac, rest was considered beneficial. In reality, the last two—namely, perfect rest and as low diet as is consistent with absolute life—are most important elements in our medical treatment; and as you never have aneurisms without artheromatous changes in the coats of the arteries, alterative remedies, as potassium iodide, become valuable, more especially when syphilis is an element in the disease. In the case before us, potassium iodide did not check the progress of the affection.

But supposing, despite the treatment referred to, the aneurism goes on growing larger and larger, protruding more and more against the chest, so that the thoracic walls and ribs are giving way before it, the tissues over it are growing thinner, and the thrill and pulsation more evident each day. The question will then come up, and is often anxiously asked by the patient, "Can nothing else be done to relieve me; to ward off this impending result, and save the life, just hanging by a thread?" This is often very difficult to answer, except when the aneurism is of a vessel so located that the main trunk may be ligated; but of course, when situated in the thoracic aorta, or arch of the aorta, this cannot be done. There remains, then, nothing to do but to put something into the sac that, mechanically, will form a clot in it. Needles, horse-hair threads, etc., have been tried with little advantage. The galvanic current alone has proved a success. You know that if two needles, connected with the poles of a battery, be placed in a dish of albumen, it will be soon coagulated. I take here two needles so connected, and by placing them into the white of an egg, you see immediately the needles become coated; and as I continue the process, a firm, strong clot is formed at the negative pole, and a small, friable one at the opposite pole. This process has been taken into consideration in the operation of electrolysis.

The mode of operating is very simple. You need two sharp platinum needles, coated with gutta percha; and after freezing the skin with ice or ether-spray, you should plunge one needle, previously connected with the galvanic battery, boldly in with a single stroke, until you feel all resistance cease. The second needle is to be introduced the same way. Thus far, it will cause but little pain; but the moment the current is turned on, the heart will give a great bound and the pulse become greatly accelerated. This should not, however, be any cause for alarm. Gradually turn on the full current and leave it on for some minutes, when the operation is completed by withdrawing your needles.

Electrolysis is only applicable when the medical treatment has been tried, and when the aneurism comes up closely to the thoracic walls; but this is so often the case that it can be frequently applied. Here is a specimen from a case in which the operation has been performed twice in this Hospital, at the urgent request of the patient. The needles were introduced, and almost immediately the operations were followed by great relief of pain and great reduction of pulsation and thrill. In a short time, however, all these bad symptoms returned, and the operation was repeated with the same beneficial results. The man finally died from exhaustion, and, at a post-mortem examination, large fibrous clots were found at the points of insertion of the needles, which, no doubt, prevented rupture of the sac. The operation, therefore, was perfectly satisfactory, so far as relief of pain and comfort are concerned. The last fact is the same as shown by the study of the results of about one hundred cases, in whom, in about two-thirds of them, temporary relief from pain and suffering was given, and only in a very small per cent. had it a curative effect; still, in no case had it ever done any harm. I think, myself, that the operation under such circumstances as I have stated, is perfectly justifiable, and I would be perfectly willing to have it performed upon myself.—*William Pepper, M.D., in Va. Med. Monthly.*

---

THE THERAPEUTICS OF VENESECTION.—From the consideration of this subject we are justified in drawing the following conclusions:

1. That although the errors of former days, without doubt, allowed a very great abuse of venesection, it has sufficient merit as a therapeutic agent to demand our earnest consideration.

2. If we are sincere in following the motto of our Society, *Natura duce*, we shall take the suggestions which nature gives and bleed in carefully selected cases.

3. That in febrile attacks a loss of blood will lower the temperature, and this decrease in temperature, is known to be disproportionate to the amount of blood lost.

4. That by venesection we do not actually diminish the volume of blood, but we cause the blood to become more watery, the free passage of the blood through the pulmonary circuit seems to be promoted, and the functional labor which the lungs have to perform is diminished by the abstraction of a certain number of the more solid particles.

5. It is fallacious to depend upon the condition of the pulse alone as the criterion of the amount of blood to be removed, or the benefit which the patient derives by a venesection. After a venesection the pulse sometimes appears to indicate increased power of the heart's action. The artery seems to strikes against the finger with more force than before the abstraction of blood. Formerly practitioners were misled by this effect upon the pulse, and blood-letting was employed as a means of increasing the power of the heart's action. The sensation which the finger receives is delusive, and is caused by the quickness of the movements of the artery. This has been shown by the sphygmograph to depend on the diminished tension of the arteries following the abstraction of blood. It is to be borne in mind, says Flint, in estimating the power of the heart's action by the sensible characters of the pulse, that the sense of resistance which is felt and the amount of pressure required to impress the artery are the evidences of strength. I cannot do better than make use of the statements quoted in Dr. Bowditch's monograph on venesection which he read at the annual meeting of the Massachusetts Medical Society, in 1871.

"Dr. Richardson says: 'If blood-letting were in this day an unknown remedy, and were some man to discover it, we should receive that man as the greatest amongst us, and send him to posterity as one of the lights of the age.' And again he says: 'The confidence of the ancients in the practice of blood-letting, their fearlessness of any immediate danger from it, was, I believe, as well founded in truth, as the cowardice and assertion, without observation, of the present day is founded on error.' He sums up as follows: 'I would recall that blood-letting as a point of scientific practice is still open to us in some stages (early stages) of typhoid fever, in cases where there is a sudden tension of blood, of which sunstroke is an example; in cases of chronic congestion of the brain; in cases of acute pain from (inflamed) serous membrane; in some cases of spasmodic pain (gall-stones, etc.); in others of sudden arrest of circulation from concussion; in congestion of the right heart, and it may be in some cases of extreme hemorrhage. Above all I claim for it a first place in the treatment of simple uræmic poisoning.'

"Dr. Sutton gives cases in which bleeding was resorted to to relieve distention of heart and passive congestion of the lungs. He ordered it, not to relieve inflammation, but to cure obstructions.

"Fordyce Barker, M. D.: 'I am gradually getting to bleed more frequently. My conviction that this resource in practice has been too much neglected by myself and others has been progressively growing for some years.' Dr. Barker would bleed to prevent abortion in some cases. So in renal congestions of the brain with coma, and when the skin is hot there is nothing so sure. He would bleed a woman in convulsions thirty ounces, and give elaterium also. 'We must not,' he also declares, 'avoid bleeding in some cases even if the patient is feeble. In puerperal mania, at least in some very rare cases, venesection is of the greatest benefit.—*William A. Dunn, M.D., in Boston Med. and Surg. Journal.*

---

PLUMBING REGULATIONS.—We copy from *The Sanitarian*, for October, the rules laid down by the Board of Health of New York for the proper performance of plumbing work, adopted under an Act of the New York Legislature, passed in 1881. We do this as we believe they will be valuable, as a reference, to not only local boards of health, but to physicians, who are frequently consulted on allied subjects.

1. All material must be of good quality and free from defects; the work must be executed in a thorough and workmanlike manner.

2. The arrangement of soil and waste-pipes must be as direct as possible, and it should run along the cellar wall unless this is impracticable, in which case it should be laid in a trench cut at a uniform grade. All changes in direction must be made with curved pipes, and all connections with Y branch pipes, and one-eighth bends. There should be an inlet for fresh air, entering the drain just inside the trap, of at least four inches in diameter leading to the outer air and opening at any convenient place not too near a window. No brick, sheet metal or earthenware flue shall be used as a sewer ventilator, nor shall any chimney flue be used for this purpose.

3. Every soil-pipe and waste-pipe must be of iron and must extend at least two feet above the highest part of the roof or coping, of undiminished size, with a return bend or cowl. It must not open near a window nor an air shaft ventilating living rooms. Horizontal soil and waste-pipes are prohibited. There should be no traps on vertical soil-pipes or vertical waste-pipes.

4. All iron pipes must be sound, free from holes, and of a uniform thickness of not less than one-eighth of an inch for a diameter of two, three or four inches, or five thirty-seconds of an inch for a diameter of five or six inches. Before they are connected they must be thoroughly coated outside and inside with coal tar pitch, applied hot, or some other equivalent substance.

5. All drums in the soil-pipes, drain-pipes, and waste-pipes must be so caulked with oakum and lead, or with cement made of sal-ammoniac and iron filings, as to make them impermeable to gases. All connections of lead with iron pipes should be made with a brass sleeve or ferule. All connections of lead pipe should be by wiped joints.

6. Every sink, basin, wash tray, bath, safe and every tub or set of tubs must be separately and effectively trap.

ped. The traps must be placed as near the fixtures as practicable. All exit-pipes should be provided with strong metallic strainers.

7. Traps should be protected from syphonage by a special metalic air-pipe, not less than one and one-half inches in diameter. If it supply air to a water-closet trap, not less than two inches in diameter, the size to increase with the number of water-closets. These pipes should extend two feet above the highest part of the roof or coping, and if independent of the upper end of the soil-pipe this extension should not be less than four inches in diameter, to avoid obstruction from frost. These air pipes must always have a continuous slope, to avoid collecting water by condensation.

8. Every safe under a wash-basin, bath, urinal, water-closet, or other fixture, must be drained by a special pipe not directly connected with any soil-pipe, waste-pipe, drain or sewer, but discharging into an open sink upon the cellar floor or outside the house.

9. No waste-pipe from a refrigerator shall be directly connected with the soil or waste-pipe, or with the drain or sewer, or discharge into the soil, but it should discharge into an open sink.

10. All water-closets inside the house must be supplied with water from a special tank or cistern, the water of which is not used for any other purpose. The closets must never be supplied directly from the Croton supply pipes. A group of water-closets may be supplied from one tank, if on the same floor and contiguous. The overflow pipes from tanks should discharge into an open sink or into the bowl of the closet itself, not into the soil or waste pipe nor into the drain or sewer. When the pressure of the Croton is not sufficient to supply these tanks a pump must be provided.

11. Cisterns for drinking water are objectionable; if indispensable, they must never be lined with lead, galvanized iron or zinc.

12. Rain-water leaders must never be used as soil, waste or vent pipes; nor shall any soil, waste or vent pipe be used as a leader.

13. No steam exhaust will be allowed to connect with any soil or waste pipe.

14. Yards and areas should always be properly graded, cemented, flagged, or well paved and drained by pipes discharging into the house drain.

15. No privy vault or cesspool for sewage will be permitted in any part of the city, when a sewer is accessible. —*New England Med. Monthly.*

---

Anæsthetics Medico-Legally Considered.—D. J. G. Johnson (*Medico-Legal Society's Bulletin*) in an excellent paper makes the following points worthy of attention:

1. Anæsthetics do stimulate the sexual functions; the ano-genital region is the last to give up its sensitiveness. Charges made by females under the influence of an anæsthetic should be received as the testimony of an insane person is. It cannot be rejected; but the *corpus delicti aliunde* rule should be insisted on. Dentists or surgeons who do not protect themselves by having a third person present do not merit much sympathy.

2. Death from administration of chloroform after a felonious assault, unless the wounding was an inevitably fatal one, reduces the crime of the prisoner from murder to a felonious assault.

3. The surgeon has no right to use chloroform to detect crime against the will of the criminal.

4. The army surgeon has the right to use chloroform to detect malingerers.

5. The medical expert, notwithstanding he is sent by order of the court, has no right to administer an anæsthetic against the wish of the plaintiff in a personal damage suit, to detect fraud.

6. Gross violations of the well-known rules of administering anæsthetics, life being lost thereby, will subject the violator to a trial on the charge of manslaughter.

7. A surgeon allowing an untrained medical student to administer anæsthetics, and life being thereby lost, will subject the surgeon himself to a suit for damages. What he does through his agent he does himself.

8. The physician who administers an anæsthetic should attend to that part of the work and nothing else. He should have carefully examined the heart and lungs beforehand. He should have the patient in the reclining position, with his clothes loose, so as not to interfere with respiration; should have his rat-tooth forceps, nitrite of amyl, and ammonia, and know their use, and when to use them and artificial respiration.

9. In operations on the ano-genital region and the evulsion of the toe nail, complete loss of sensation in these parts should never be allowed, and no operation on these parts at all should be had under an anæsthetic unless by the approval of a full consultation, who have a knowledge of the dangers.

10. Chloroform cannot be administered to persons who are asleep without waking them. Experts themselves, with the utmost care, fail more often than they succeed in chloroforming adults in their sleep.—*Detroit Lancet.*

---

BAD EFFECTS FROM THE INORDINATE USE OF IODOFORM.—The application of large quantities of powdered iodoform to granulating surfaces does not seem to be as harmless as has been hitherto supposed. Since the recommendation by Mikuliez, German surgeons have applied iodoform in large quantities to the wounds caused by resections and carious cavities, without untoward results. But two deaths are now reported by Dr. Henry, (*Deutsche Medicinische Wochenshrift*, No. 34, 1881), not referable to anything but iodoform poisoning. One was an extended resection of the elbow, on account of fungous synovitis, with intra-muscular abscesses, in a man fifty-seven years of age. After the operation the entire cavity was packed with about one hundred and fifty to two hundred grammes of iodoform, a quantity no larger than that has often been employed. The operation had been performed antiseptically. The patient became somewhat excited and even delirious within a day, subsequently remarkably quiet. He stayed in bed with open eyes, indifferent to his surroundings, and evidently misunderstanding any questions asked him. Food was taken when handed to him, and the scanty urine was passed in bed. This state increased. The temperature remained normal, but the pulse was frequent and small. The sinking in of the abdomen and stiffness of the occipital muscles gave the appearance of tubercular meningitis. The patient died on the fifth evening in deep coma, with symptoms of pulmonary œdema. The only anomalies, of consequence, revealed by the post-mortem, were fatty degeneration of the heart, kidneys and liver. A second case died under similar circumstances, with the same symptoms and lesions. Cerebral depression and muscular weakness are the symptoms produced by iodoform poisoning in animals. When these appeared the dressing was removed, but without stopping the course of the poisoning. The urine was diminished in quantity and contained no albumen during life, but large quantities of iodides. The author advises caution in the use of large quantities of the substance, especially in old and feeble individuals.—*New England Med. Monthly.*

---

Dilatation in Phimosis.—Prof. Verneuil (*Gaz. des Hop.*)—When it is characterized by mere narrowness of the orifice of the prepuce, a *debridement* carried parallel to the axis of the prepuce, either by means of scissors, bistoury or elastic cord, will suffice. It is a good operation when the narrowing is especially due to the formation of cicatricial tissue, as is observed, for example, after chancre. It is sufficient then to make the *debridement*, and leave the parts to themselves. There is also circumcision—an operation far more difficult than is usually supposed. Sometimes it succeeds very well, but most frequently union does not take place by first intention, and the results of the operation are very prolonged. In some cases, too, it gives rise to more or less hæmorrhage, and in others to more or less dangerous consequences. Prof. Verneuil resorted to dilatation, which he has now performed for a long time, reserving circumcision for special cases, even in phimosis produced by cicatricial contraction, except in the very rare cases in which the tissue is very hard. Special forceps have been invented, but in country practice the common dressing forceps are just as convenient.

The operative procedure is very simple. I put my patient to sleep because I wish to proceed as slowly as necessary, and to obviate the pain inherent to the operation. I draw out the prepuce, and commence by introducing a grooved director between the prepuce and the glans, and then I pass a second grooved director along the groove of the first. In this way a commencement of dilatation takes place, and then I introduce a common dressing forceps, open it and withdraw gradually, distending the prepuce, just as the anus is dilated by a speculum. I have never yet met with a failure. All that can happen is a slight rupture of the preputial mucous membrane, giving rise to a few drops of blood. When the prepuce thus dilated is everted, you wash the glans with some carbolized water. If the dilatation has been very considerable, you then close the prepuce over the glans. If not, you will have a paraphimosis, which you should dress with lead lotion, without any fear that gangrene of the glans or the penis will be produced by strangulation. Still, gangrene of the glans does occasionally take place. *Med. Times and Gazette.—Gaillard's Med. Journal.*

---

Professional Earnings in England.—We have recently allotted special space to the notification of wills left by medical men. It must have already struck those of our readers who have glanced at the figures recorded in this weekly report, that the average value of property handed down by members of the profession to their families is singularly small. This is, unhappily, the fact. The general practitioner is a hard-working and too often a struggling man to the end of his days. Comparatively few of the members of the class are able to retire, as the members of other callings retire, for rest from their labors, before the relief which brings rest to all men. Physicians and surgeons as a rule die in harness. The expenses incurred by those who make specialties of medicine or surgery, or of any one branch of either of those departments of professional work, are necessarily great, while the recompense to the life of labor entailed, looking at the career as a whole, is proportionately small. Even the few who seem to be making large incomes during a part of their career seldom amass even moderate competencies. Some five-and-twenty years ago calculations were made for London and the provinces, and it was estimated that a physician, practicing as such in London, did not acquire an income on which he would be required to pay income-tax for sixteen years from the commencement, while a physician in the provinces reached the legal figure in eleven years, but not earlier. This difference in favor of the provinces is, of course, due to the fact that no man would think of commencing practice as a pure physician in any city or town, except the capital, unless he had special reason to believe there existed an "opening."

We have no means of knowing whether matters have mended with the profession generally during the last quarter of a century, but looking to the increase of its aggregate numbers in relation to the increase of population, we fear there is not much ground to hope that the rewards of professional labor have been sensibly augmented. The laborer is worthy of his hire, and it is well now and again to look into the matter of money. It will sooner or later be necessary to take it into very serious consideration in relation to the question of fees. Meanwhile the lesson to be learnt from the story of the wills left by medical men is certainly one of caution and thrift. It is a sad reflection that, speaking generally, the families of medical practitioners are insufficiently provided for, a large proportion being left almost in poverty.—*The Lancet.* —*Med. Gazette.*

---

Why are not Medical Societies more Prosperous?—The Buffalo *Medical Journal* says that at a recent meeting of the Medical Association of that city, it was announced that an able gentleman would read a paper upon the relation of germs to the etiology of disease. At this meeting a quorum failed to be present. At an adjourned meeting less than a dozen were present. The *Journal* thinks this is a proof that the medical profession is not interested in scientific questions. The fact is, as it seems to us, about this. To secure attendance upon the meetings of a medical society requires constant effort on the part of some one or more persons. Skillful plans well executed will make a medical society prosperous. The difficulty is to find one who will take the trouble to devise such plans and see that they are executed. For real medical work, small societies, meeting frequently, in a semi-social manner are demonstrably the most successful.

For purposes of medical politics larger organizations are needful, and are governed by different laws. The tendency of the medical profession is toward disintegration rather than toward aggregation. This is doubtless owing to the nature of the physician's work. Mainly this must be done entirely alone. Then he is so often disturbed by dishonorable or unwise associates in the matter of council or otherwise, that he finds an isolated life the most peaceful and the most profitable. To break over this habit of distrust of medical men in general, and abandon his peaceful life, for one of more or less constant conflict, requires, generally, some inducements greater than the annoyances and the troubles attending these organizations. These, it seems to us are only to be found in either real instruction or in pleasant association. If there be a certainty of both instruction and pleasant association, then there is a double force drawing him to attendance upon the meetings of medical societies and actual work for the same. Again, if every encouragement is given for active contributions on the part of each member to each meeting, his attendance is likely to be more sure.

But, as we remarked previously, to secure these ends requires some organizing person who is willing to do this service for the others.—*Detroit Lancet.*

---

NOTE ON THE SANITARY CONDITION OF BAGSHOT PARK.—It has been suggested to me by His Royal Highness the Duke of Connaught, that, as a matter of general interest to the medical profession, I should communicate a brief notice of the results of the careful investigation and report made by Mr. Rogers Field into the sanitary condition of Bagshot Park, the defects in which are believed to have led directly to the Duchess of Connaught's late serious illness, from which she is now happily entirely convalescent. This is a suggestion I willingly comply with, in the hope that so prominent an instance of the danger that may arise from such faults of construction and workmanship as have been proved to exist may have the effect of directing attention to the paramount importance of questions of this kind.

It may be well to premise that the present house at Bagshot Park is not that long occupied by the late Sir James Clark, but an entirely new building, recently erected at a cost of from thirty to forty thousand pounds. As a matter of course, considerable pains were taken in the arrangement and ventilation of the drains; but it appears that, not only was the system adopted in itself defective, but that the work was in many instances so carelessly carried out, that it is a matter of astonishment that more grave results have not followed.

A few examples of the defects discovered will suffice to show what was really the state of the sanitary arrangements. "The drains, taking certain baths and sinks, run underneath the house, with two chambers in their course, likewise underneath the house. Connected with these bath drains are several land drains, constructed of ordinary jointed pipes. This, I need hardly point out, is a very objectionable arrangement, as the bath drains are in unbroken communication with soil drains, the unventilated trap forming no reliable security against the passage of foul air."

All the drains inside the house, and immediately around the house, "were found, without exception, to be leaky, some of them exceedingly so."

On testing the drains, taking the soil pipe from one closet, "water ran out of the pipe through the wall into the basement, and, on opening up the pipe just outside the wall, it was found to be not only badly jointed, but broken, and there was an accumulation of filth which had run from it into the ground."

"The bath and sink drain under the house was so completely stopped up with a slimy deposit, that it was some time before any water could pass down it."

"On examining the basement on his first visit, my assistant noticed a vertical lead pipe communicating directly with the soil drains. On inquiry he was informed that this pipe was intended for a sink which had not been fixed; and he was assured by the clerk of the works that the upper end of the pipe had been cut off and carefully sealed up. It was afterwards discovered that there had been no attempt at sealing up the pipe, but that the upper end had been left open immediately under the floor of the vestibule, and thus afforded a free passage for foul air into the house."

"All of the closets had a disagreeable smell, and some of them were exceedingly offensive."

"The waste pipes of the baths are in unbroken communication with the drains, instead of discharging with open ends outside the house, as they should do."

"The waste pipes of two of the sinks have unbroken connections with the drains in the basement"

The following is specially interesting under the circumstances which led to this investigation: "In order to ascertain how far the smell from the leaky drains and rain water overflow in the basement would penetrate, I applied the smoke test, by burning specially prepared paper in the basements under the central saloon, study, waiting room and vestibule. The smoke was seen to issue in great volume from the hot water coils in the saloon, and a strong smell of smoke was perceived in the study, waiting room and vestibule. It was also proved that the smoke passed from the saloon into the Duchess's bedroom. Again, on burning the special paper in the hollow wall at the back of the heating furnace, the smoke found its way into the dining room, up the casing of the soil pipe into the Duke's dressing room, and thence into the Duchess's bed room. From the foregoing tests, it will be seen that there were two distinct and easy ways by which the foul gases from the drains in the basement could find their way into the Duchess's bed room."

The whole question of the effects of defective sanitary condition on the puerperal state is one which deserves much more serious study than it has yet received. I have long been satisfied that they have often much to do with grave forms of illness in the lying-in state, the origin of which cannot otherwise be traced. In the chapter on "Puerperal Septicæmia," in the third edition of my work on *The Science and Practice of Midwifery*, I wrote as follows: "Exposure to sewer gas may, I feel sure, produce the disease. In two cases of the kind I had the opportunity of closely watching, an untrapped drain opened directly into the bedroom, in one instance into a bath, in the other into a water closet. Both cases were indistinguishable from the ordinary form of the disease, and in both improvement commenced as soon as the patient was removed into another room."—*W. S. Playfair, M.D., F. R. C. P., in Sanitary Journal, Glasgow.*

---

"STAMMERING."—1. We call the attention of our pupils to the fact that they must apply themselves seriously and with perseverance to practice our system, until it becomes a settled habit with them.

2. Before speaking they must be careful to take a full and quiet breath and to renew their respiration according to the sense of the phrase, and never to speak when the air is exhausted.

3. Put into practice the observations made relative to the movements of the lips and tongue.

4. Preserve a good syllabation. This is easily hidden by the intonations and inflexions of the voice.

5. Speak with assurance, watch over the emission of your words, exercise full control over yourself, and the more you feel embarrassed the more you must speak slowly, coolly and deliberately, in a word be ever on your guard and watch yourself attentively.

6. We may sum up our system in three words: Respiration, syllabation and tranquility. These include everything and they are equally indispensable.

7. Take advantage of all opportunities to speak slowly, as for instance when you are with your family or friends. —*Prof. Delon in Cin. Lancet and Clinic.*

---

Oophorectomy.—A test of the estimation in which this operation is held by the various gynæcologists is to be found in the number of operations reported by each. Dr. Battey, the originator of the operation, whose conservatism is well known, has performed the operation but sixteen times in the course of ten years; Mathews Duncan but once. Spencer Wells regards the operation as being but very infrequently necessary, which is also the opinion of Martin, of Berlin. Lawson Tait, of Birmingham, England, has, on the other hand, performed oophorectomy no less than sixty-five times in less than two years. The contrast between the infrequency with which Battey has performed the operation as compared with the great number of cases in which Lawson Tait has found it necessary, is somewhat remarkable.—*Chicago Med Review.*

---

Antiseptic Midwifery —Dr. Robt. Barnes, in a paper "On Antiseptic Midwifery and Septicemia in Midwifery" (*Amer. Jour. Obstetrics*, Jan., 1882), summarizes in the following rules: 1. Keep the door shut against the enemy by maintaining contraction of the uterus. 2. Prevent the enemy from forming and collecting by irrigating the parturient canal with antiseptic fluids. 3. Eject the enemy as fast as he effects an entry; that is, keep the excretory organs in activity. 4. Guard the lying-in chamber against the approach of foreign poisons. 5. Fortify the patient against the attack of the enemy by keeping up due supplies of wholesome food.—*Maryland Med. Journal.*

---

The Use of Nitrous Oxide Gas in Labor.—Dr. Klikowitsch (*Meditz. Obozrenie*, xv., p. 759) has used this gas instead of chloroform in twenty cases of labor. He considers it much superior to chloroform or ether. Four to five inhalations are sufficient to abolish sensation without affecting consciousness, and its use is free from the objectionable after effects peculiar to the above named anæsthetics. It is said that the inhalation can be carried out under the direction of an intelligent nurse, so that the interposition of the physician is not absolutely necessary. *Med. Record.*

